# Case report: systolic murmur associated with pulmonary embolism

**DOI:** 10.1186/s12245-019-0250-y

**Published:** 2019-11-06

**Authors:** Cain M. Dudek, Kelly R. McCracken, B. James Connolly

**Affiliations:** 1 Temple University, 1801 North Broad Street, Philadelphia, PA 19122 USA; 2 Evangelical Community Hospital, 1 Hospital Drive, Lewisburg, 17837 PA USA

**Keywords:** Pulmonary embolism, Systolic murmur, Acute, Emergency medicine

## Abstract

**Background:**

This case study’s novelty lies in the potential to link a new sign in pulmonary embolism diagnosis which does not increase cost but could lead to more rapid treatment. Early intervention in these cases is vital to decrease morbidity and mortality.

**Case presentation:**

An otherwise healthy 20-year-old female patient presents to the emergency department for evaluation of a syncopal episode which occurred just prior to arriving to the emergency department. Patient also complains of ongoing shortness of breath while performing activities of daily living for 3 weeks. In this patient with no known valvular disease, physical exam revealed a systolic murmur heard only posteriorly. Subsequent emergency department workup revealed bilateral massive pulmonary emboli.

**Implications:**

A new flow murmur heard in atypical locations could be an early sign to aid in the detection and diagnosis of pulmonary embolism. This is especially important in rural community hospitals with limited access to advanced imaging modalities.

## Background

Acute pulmonary embolism (PE) is associated with high early mortality rate of up to 30%. Even with pioneering medical advances, this has not substantially changed [1]. While there are no exact epidemiological studies indicating the prevalence of pulmonary embolism cases, an estimated 300,000 to 600,000 patients are diagnosed per year with a thromboembolic event. Estimates indicate about 80,000 of these patients will die. The first symptom of pulmonary embolism is sudden death in about 25% of patients [2]. This is especially important in rural or critical access settings where catheter-directed tPA therapy is not readily available. The setting for the case is in a rural community hospital emergency department in the USA during June 2019. This case study is being reported in accordance with the case report (CARE) guidelines.

## Case presentation

A 20-year-old Caucasian female presented to the emergency department of a rural community hospital with shortness of breath and a syncopal episode. The patient stated she is a cross-country runner and for the past 4 days has had significant shortness of breath with even mild exertion. The patient had a witnessed syncopal episode at home lasting about one minute. The patient remembered being in the kitchen, but not falling. At that point, her mother brought her to the emergency department for further evaluation.

Upon arrival, the initial set of vital signs were blood pressure 132/78 mmHg; heart rate 109 bpm; regular, respiratory rate of 20; and SpO2 of 89% on room air. The patient appeared well nourished, but mildly anxious. Further assessment revealed no past medical history, no pertinent surgical history, and the only medication she takes daily is birth control. The patient endorsed recent flights from Georgia to Florida to Puerto Rico in the past 4 months. The patient denied ever feeling severe shortness of breath and denies a history of syncope. Patient’s mother denied any congenital defects or pertinent genetic information including family history of clotting disorders or history of blood clot in any first-degree relatives. While talking, the patient’s work of breathing increased as evidenced by accessory muscle use and SpO2 decreases to 89%. When resting, her SpO2 is 95–96% and showed no signs of accessory muscle use or tachypnea.

**Fig. 1:**
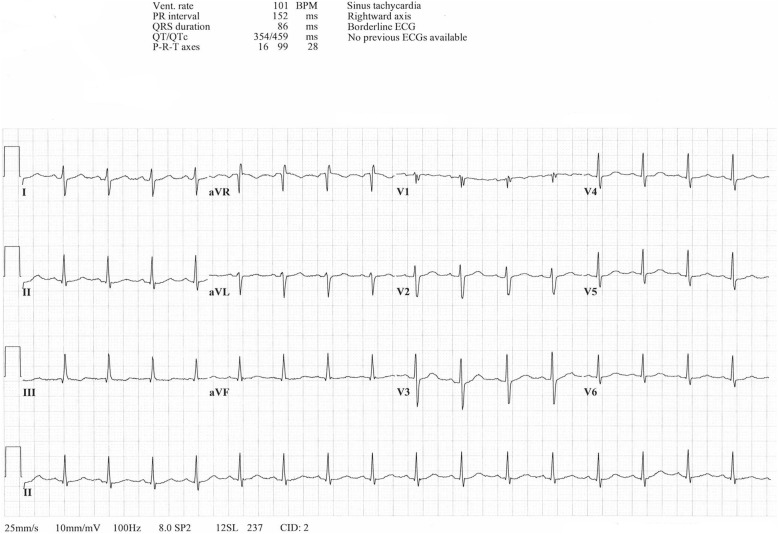
Twelve lead ECG recording while resting in the bed

Physical exam revealed clear and equal lung sounds anteriorly, heart sounds were normal anteriorly, skin was pink, warm, and mildly diaphoretic. Posteriorly lung fields were auscultated as clear, but a low-pitched systolic murmur was heard in the 5th intercostal space in the right scapula region. Lower extremities were normal upon exam. Diagnostic investigation revealed significantly elevated troponin T high sensitivity at 82 ng/L (normal < 14 ng/L). Other lab work was normal. ECG can be seen in Fig. 1 below. Pulmonary embolism was suspected and a diagnostic CT angiogram (CTA) of the chest was obtained. A d-dimer was not included in diagnostic workup because of the high index of suspicion for pulmonary embolism given the other test results.

**Fig. 2:**
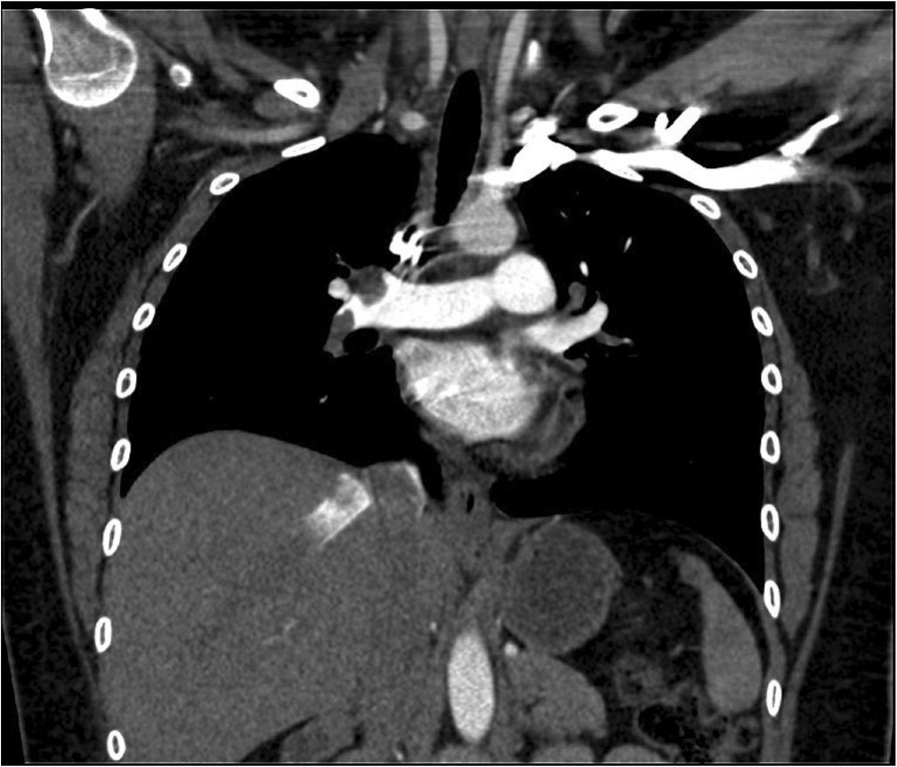
Large obstruction of right pulmonary artery at the first bifurcation

**Fig. 3:**
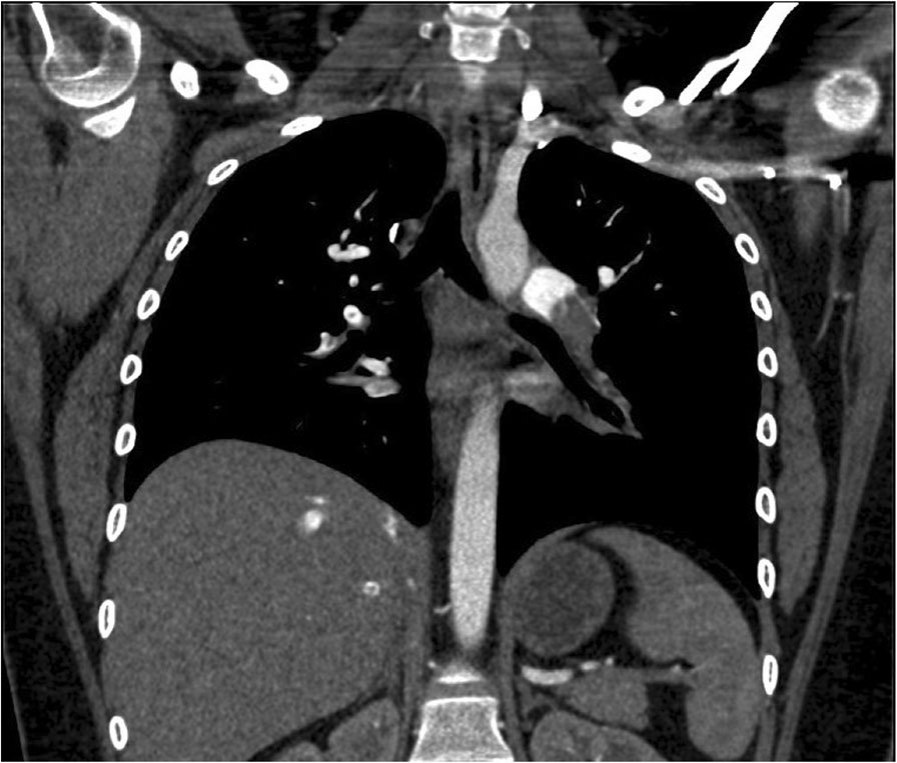
Large obstruction of the left pulmonary artery at the first bifurcation

**Fig. 4:**
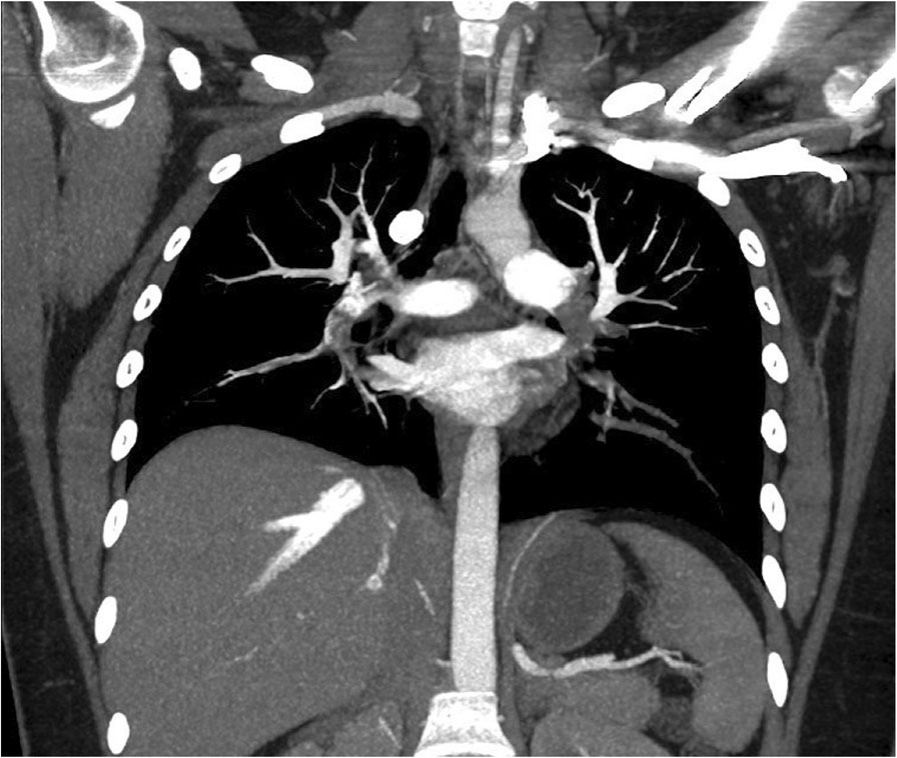
Illustrates the extensive bilateral pulmonary emboli present

The CTA of the chest was read as extensive bilateral pulmonary emboli with evidence of right heart strain, as demonstrated by refluxing of contrast into the right ventricle, see Figs. 2, 3, and 4. The patient was placed on a heparin drip and transferred to a higher acuity facility for catheter-directed thrombolytic therapy and further evaluation.

At the higher acuity facility, echocardiogram showed a mildly dilated right ventricular cavity with moderately reduced function and diffuse hypokinesis. Venous doppler studies of the right and left lower extremities were negative for thrombosis. The patient underwent radiological insertion of a catheter into the pulmonary artery for catheter-directed tPA therapy. After the catheter was removed, an IVC filter was inserted. Patient was anticoagulated and sent home after 4 days in the intensive care unit.

## Discussion

A small number of case reports have reported a systolic murmur in the context of pulmonary embolism with right heart strain [3]. We present this case report illustrating an otherwise healthy 20-year-old female exhibiting a systolic murmur with confirmed bilateral pulmonary embolism by a CT angiogram of the chest without evidence of valvular disease on echocardiogram. The likely source of the flow murmur, heard only posteriorly, was turbulent pulmonary arterial flow in the posterior segments, caused by clot burden. We believe this case in addition to other previous published cases reinforces the association between massive pulmonary embolism and a systolic murmur. A new murmur such as this could be of critical importance in the presentation of acute pulmonary embolism, especially in the setting of a patient who is unable to undergo diagnostic imaging. Faced with diagnostic uncertainty in an acutely ill patient, this finding may serve as an additional piece of information to aid the clinician in determining whether the patient may be a candidate for thrombolytics for suspected PE, especially in the deteriorating patient whom further diagnostic studies may not be obtainable. Patients presenting with a new systolic murmur and other pulmonary symptoms should have increased suspicion for massive pulmonary emboli. More case studies and further research are needed to determine the efficacy of this murmur for diagnostic purposes.

## Limitations

This case report does have some weaknesses. We were unable to record the murmur due to a lack of necessary equipment. Even with these limitations, we believe there is strong evidence to support a link between the systolic murmur and the pulmonary embolism.

## Data Availability

The data generated and analyzed for this case study are publicly available and included in the references section of this article.

## Abbreviations

CT: Computed tomography
CTA: Computed tomography angiogram
PE: Pulmonary embolism
